# SpiFoG: an efficient supervised learning algorithm for the network of spiking neurons

**DOI:** 10.1038/s41598-020-70136-5

**Published:** 2020-08-04

**Authors:** Irshed Hussain, Dalton Meitei Thounaojam

**Affiliations:** Computer Vision Laboratory, Department of Computer Science and Engineering, National Institute of Technology Silchar, Silchar, Assam 788010 India

**Keywords:** Computational science, Computer science

## Abstract

There has been a lot of research on supervised learning in spiking neural network (SNN) for a couple of decades to improve computational efficiency. However, evolutionary algorithm based supervised learning for SNN has not been investigated thoroughly which is still in embryo stage. This paper introduce an efficient algorithm (SpiFoG) to train multilayer feed forward SNN in supervised manner that uses elitist floating point genetic algorithm with hybrid crossover. The evidence from neuroscience claims that the brain uses spike times with random synaptic delays for information processing. Therefore, leaky-integrate-and-fire spiking neuron is used in this research introducing random synaptic delays. The SpiFoG allows both excitatory and inhibitory neurons by allowing a mixture of positive and negative synaptic weights. In addition, random synaptic delays are also trained with synaptic weights in an efficient manner. Moreover, computational efficiency of SpiFoG was increased by reducing the total simulation time and increasing the time step since increasing time step within the total simulation time takes less iteration. The SpiFoG is benchmarked on Iris and WBC dataset drawn from the UCI machine learning repository and found better performance than state-of-the-art techniques.

## Introduction

The organic motivation of massive parallel human brain drive researchers towards the field of innovation such as spiking neural network (SNN). Evidence from neuroscience proves that the actual information processing inside the brain utilise precise timing of spikes rather than the average firing rates^1^. SNN uses precise timing of spikes for information processing which is considered as the third generation of neural network^2^. It is computationally more powerful due to the capability of solving the same classification problem with less number of neurons than that of second generation artificial neural network (ANN)^3^. In addition, SNN is biologically plausible, energy efficient and has the capability to work with neuromorphic hardware efficiently^4, 5^. The spiking neurons in SNN communicate with each other by sending a signal characterised by sudden increase in voltages called action potential or spike^3^. The real valued input features need to be encoded into discrete events of precise timing of spikes in order to work with SNN. The encoding method used by biological neuron is not clear to date^6, 7^. However, most of the researchers uses population encoding^5, 8^ to carry out experiment using SNN. According to the investigation of encoding scheme, the first spike carries more relevant information than the later spikes and that can be achieved by the time-to-first-spike encoding scheme proposed in^8^. Note that a spiking neuron does not fire spikes at each propagation cycle, rather it follows the mechanism of information processing akin to the brain that fires spike only when the membrane potential crosses a threshold. The spikes are sent to the receiving neurons (i.e., post-synaptic neurons) by the sending neurons (i.e., pre-synaptic neurons). Thus the membrane potential of a post-synaptic neuron called post-synaptic potential (PSP) changes upon receiving the inputs from pre-synaptic neurons. Post-synaptic neuron fires a spike when its membrane potential reaches a threshold. Typical values of resting membrane potential and threshold in biological neuron are around − 70 mV and − 55 mV respectively^9^. When neuron send spikes to other neuron there is a delay called synaptic delay in information transmission due to the length of axon cable and other factors such as noise. Experimental evidence proves the presence and role of synaptic delay in biological neurons^10–12^. The dynamics of a spiking neuron is characterised by its neuron model. The leaky-integrate-and-fire (LIF)^13^ neuron model which is an improved version of integrate-and-fire^14^ spiking neuron, is simple and has good computational efficiency. Note that, the association of synaptic delay with LIF is less explored. In this paper, LIF model is experimented with delays in an efficient manner. There has been a lot of attention towards the development of an efficient supervised learning algorithm for SNN to train the network in computationally efficient manner as well as to add biological plausibility for a couple of decades. Bohte et al.^8^ introduced a supervised learning algorithm for multilayer feed forward SNN called SpikeProp akin to the back-propagation algorithm. In SpikeProp algorithm, time-to-first-spike input encoding scheme was used to overcome the problem of discontinuity i.e., input, hidden and output neurons can fire only a single spike and the spikes after the first spike are discarded. Later, it has been found that although Bohte et al.^8^ used a small value of learning rate, a large value of learning rate can also lead to a successful training^15^. To speed up the convergence rate, performance and to allow multiple spikes, SpikeProp is further investigated in^15–20^. The demerits of gradient rule based learning is the surge or sudden jumps, and discontinuities in the error function as discussed by Shrestha et al.^16^. Sporea et al.^21^ had used spike time dependent plasticity (STDP) and anti-STDP to device more similar biological supervised learning for multilayer SNN. This method allows neuron in all the layers to fire multiple times. However, authors did not consider hidden neurons spike time while training, which is a barrier to the biological plausibility. In^22^, a survey on supervised learning for SNN is carried out where various supervised learning methods was compared. Quiang et al.^23^ proposed another supervised learning algorithm that uses temporal encoding. Ponulak et al.^24^ proposed remote supervised method (ReSuMe) and argued that the method possesses interesting properties of the supervised Hebbian approach while avoiding its drawback. The main advantage of ReSuMe method is that the method is independent of the neuron models used and can be used in a variety of neuron models. In extended delay based ReSuMe^25^, and in DL-ReSuMe^12^, a delay learning based remote supervision method is investigated for the network of spiking neurons. Note that in^12, 25^, constant synaptic delays was used. In^26^, multiple neurons are trained instead of a single neuron. Aboozar et al.^27^ proposed BPSL algorithm that can fire multiple desired spikes from a single neuron. John et al.^28^, proposed another STDP based supervised learning algorithm SWAT. In^29^, a supervised training algorithm is proposed where a neuron learns spike timing-based decisions called tempotron which allows neurons to learn whether to fire or not in response to a particular input stimuli. However, in tempotron neuron did not learn precise desired output spike train, rather decides the firing ability of the neuron. Ammar Mohemmed et al.^30, 31^ developed a supervised learning algorithm using extended delta learning rule where each spike sequence is convolved with a suitable kernel function. J. Wang et al.^32^ proposed OSNN algorithm having adaptive structure based supervised learning ability. There exist membrane potential driven learning algorithms proposed for SNN such as MPD-AL^33^, EMPD^34^, and MemPo-Learn^35^. In the development of MPD-AL^33^ algorithm, two approaches were used namely firing less spikes than desired and firing more spikes than desired. In^34^, two process namely at desired output times and at undesired output times were used. The error function proposed in^35^ is formed by taking difference between membrane potential of output neuron and the firing threshold of its own. The ability of evolutionary strategies (ES) to work directly with real numbers without complex encoding technique drives researchers towards the investigation of ES to train SNN. In^36, 37^, SNN was trained using evolutionary techniques. Evolutionary methods also had been used to optimise SpikeProp in^38^ where particle swarm optimization (PSO) technique was used. In^37^, a self regulating evolving SNN named SRESN was proposed. Differential evolution is also used to develop a supervised learning algorithm for SNN in^39^ called DEPT-ESNN. The limitation of ES based learning algorithm is that these algorithm demands the neuron in SNN to fire exactly once. Therefore, ES technique cannot be used efficiently where there is multiple spiking in SNN. In addition, ES based learning technique is very time consuming.

There is an existing gap between neuroscience and machine learning to date. Although SNN is well capable to bridge this gap, but remained unexplored for decades as it was considered too complex to implement practically and difficulty in analysing the performance. Apart from that, adding biological plausibility is a much bigger challenge in terms of computational cost. In addition, due to the lack of efficient supervised learning algorithm and difficulty in software implementation, SNN became less popular although it is energy efficient, provides good computational power and biologically plausible. Moreover, SNN is very challenging due to its discrete spiking nature. The biological plausibility of unsupervised learning such as STDP, long term potential (LTP) and long term depression (LTD) in SNN is investigated and used by many researchers over the last couple of decades^5, 40^. However, unsupervised learning paradigms are not suitable for classification problems and the classification of spatio-temporal pattern becomes very essential in the current scenario. Also from literature review it is found that the development of an efficient supervised learning algorithm for SNN is still in its infancy stage. Therefore, this paper presents an efficient algorithm to train multilayer Spiking neural network which uses elitist Floating pointGenetic algorithm (SpiFoG).

The main contributions of this paper is as follows:

1. An efficient supervised learning algorithm (SpiFoG) for multilayer feed-forward SNN is proposed.
2. Random delays are introduced in the synapse model of LIF neuron which are also trained with synaptic weights.
3. Both positive and negative values are allowed to add more biological plausibility to the system.
4. The total simulation time is reduced, and the value of time step is increased to improve computational efficiency.
5. Hybrid crossover method is used for the faster convergence providing well exploration of the search space.

## Results

To evaluate the performance, SpiFoG is benchmarked against the Iris (multiclass) and WBC (binary) dataset drawn from the UCI machine learning repository. For every dataset, the experiment is repeated 10 times randomly.

### Dataset description

The Iris dataset^41^, represents three classes such as *Setosa* (Class 1), *Versicolor* (Class 2), and *Virginica* (Class 3). There are 150 instances each having 4 real valued features. These real valued features are mapped into spike times to yield 16 features (4$\times L$). In the dataset, each class has 50 instances. In order to do a better comparison with the state-of-the-art algorithms, we have used the same number of training and testing samples as taken by the state-of-the-art algorithms. Therefore, for training, 50% of 150 instances are used and rest are used to test the performance. The WBC dataset^42^, represents two classes such as *Benign* (Class 1), and *Malignant* (Class 2). There are 699 instances with 16 missing values. For training, 333 instances are used and for testing 350 instances are used akin to SpikeProp algorithm^8^.

### Performance measures

The performance of the SpiFoG is evaluated in terms of Accuracy (Training set and Test set), and computational efficiency (CE) defined in the Eq. (1). Table 1 shows the comparison in terms of mean accuracy along with standard deviation, and in terms of CE which is a better way to compare among the algorithms since running time in different machine with different configuration is different. The more the value of CE, better is an algorithm.

1 $$\begin{aligned} CE = \frac{dt}{T \times G} \end{aligned}$$

where *G* is the epoch at which the algorithm converges and it is independent of total simulation time *T* (it is the maximum time at which a neuron must issue atleast a single spike and to do that efficient setting of parameters are very necessary). There is no correlation between *G* and *T*, former is related to tuning parameters at training phase and later is related to issuing spikes after which the tuning is possible.

**Table 1:** The comparison of results for SpiFoG algorithm with the state-of-the-art techniques.

Dataset	Algorithm	Time step (dt)	Simulation time (T)	Epoch (G)	Accuracy (%)		CE
					Training set	Test set	
Iris	SpikeProp^8^	0.01	50	1,000	97.4 ± 0.1	96.1 ± 0.1	0.20$\times 10^{-6}$
	SWAT^28^	–	–	500	95.5 ± 0.6	95.3 ± 3.6	–
	PSO-SpikeProp^38^	0.01	50	231	72.27	84.66	0.87$\times 10^{-6}$
	SRESN^37^ (online)	–	–	**102**	92.7 ± 4.2	93.0 ± 5.7	–
	DEPT-ESNN^39^	–	–	–	$\mathbf {99.33} \pm \mathbf {0.24}$	89.33 ± 3.44	–
	SpiFoG	**1**	**20**	299	97.41 ± 0.89	$\mathbf {97.24} \pm \mathbf {2.12}$	$\mathbf {0.12} \times \mathbf {10^{-3}}$
		0.1	**20**	503	96.95 ± 0.23	97.06 ± 0.48	0.99$\times 10^{-5}$
	SpikeProp^8^	0.01	50	1,500	97.6 ± 0.2	97.0 ± 0.6	0.13$\times 10^{-6}$
	SWAT^28^	–	–	500	96.2 ± 0.4	96.7 ± 2.3	–
WBC	PSO-SpikeProp^38^	0.01	50	**232**	63.82	62.7	0.86$\times 10^{-6}$
	SRESN^37^ (online)	–	–	306	93.9 ± 1.8	94.0 ± 2.6	–
	OSNN^32^	–	–	–	91.1 ± 2.0	90.4 ± 1.8	–
	SpiFoG	**1**	**20**	896	97.97 ± 0.48	96.81 ± 0.84	$\mathbf {0.55} \times \mathbf {10^{-4}}$
		0.1	**20**	597	$\mathbf {98.32} \pm \mathbf {0.32}$	$\mathbf {97.92} \pm \mathbf {0.13}$	0.83 $\times 10^{-5}$

Bold indicates the best results.

## Discussion

The selection of hyper-parameters such as $dt, T, \tau _{m}, \tau _{s}, R_{m}$ in an efficient manner is very essential and necessary in case of SNN since each one is somehow related to each other whether it is the production of spikes or in training phase. A learning algorithm can only tune parameters to reduce overall network error after the production of spike times otherwise there is no way to compute the value of error. In our case, the selection of few parameter effects the performance of SpiFoG both in terms of accuracy (training set and test set) and CE. In this experiment, the total simulation time denoted by *T* is set to 20 ms which is less than state-of-the-art techniques and it is directly responsible for providing better CE. The problem with in the reduction of the value of *T* is that we have to ensure that within that time neurons fire atleast a spike and it is directly dependent on hyper-parameter setting. On the other hand, due to the efficient synaptic weight distribution, the neurons needs less iteration to fire a spike. Time step *dt* is taken as 1 ms i.e., at a step size of 1 ms each neuron updates the membrane potential that provides more speed and thereby CE increases. But, if the parameter settings are not good such as initialisation of random weights then whatever may be the value of time step there is a very less chance for a neuron to fire within the value of *T*. Therefore, before training, the setting of the range for synaptic weights is needed to be done providing a good trade off between the threshold value and the average width of the distribution. A very low value of synaptic weight results in no firing of the neurons and a very high value of synaptic weight results in early firing of the neurons. The computation of error is not possible if the neurons do not fire at all and early firing forces the algorithm to convergence prematurely. Therefore, to make the algorithm effective we have chosen the values of synaptic weights from uniform distribution within the range ${\mathbf {U}}$(-0.25, 1) allowing both positive and negative values and a mixture of inhibitory and excitatory neurons to make the system more similar to the biological neuron.

**Figure 1:**
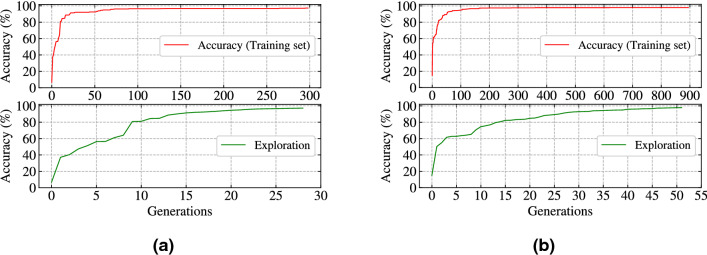
(**a**) The training accuracy up to the generation at which the algorithm convergences (top) and the search space exploration behaviour (bottom) in case of Iris dataset. (**b**) The training accuracy up to the generation at which the algorithm convergences (top) and the search space exploration behaviour (bottom) in case of WBC dataset.

In this experiment, random synaptic delays are introduced with LIF model having values from random integer distribution within the range (1, 6) which is also trained along with synaptic weights. The selection of minimum and maximum values of delays effect the speed of the algorithm. A very high value of delay unnecessarily delays the firing of a post-synaptic neuron and thereby provides a poor value of CE. There are other parameters responsible for the efficiency of SpiFoG such as double decaying kernel function, hybrid crossover method (discussed in “Methods” section) which provides better convergence of the algorithm. The kernel function having double decay was used for the experiment and the value of time constants $\tau _{m}$ and $\tau _{s}$ effects the change in membrane potential. We have chosen the value of $\tau _{m}$ as 10 ms and the value of $\tau _{s}$ as 5 ms. A small value of $\tau _{m}$ and $\tau _{s}$ results in a narrow shaped PSP and in that case, a high weight value is required to reach the threshold value as well as faces a problem of non overlapping of PSPs from pre-synaptic neurons. On the other hand, higher value of $\tau _{m}$ and $\tau _{s}$ results in a wide PSP and in that case, internal state of a neuron increases very fast which takes very long time to come to the resting potential as well as the overlapping of PSPs occur. From the experimental point of view, we have seen a value of $\tau _{m}$ equal to or slightly greater than $\Delta T$ and a value of $\tau _{s}$ as half of $\tau _{m}$ adds more stability to the shape of PSP. The utilisation of hybrid crossover leads the algorithm towards faster convergence although the search space is well explored.

**Figure 2:**
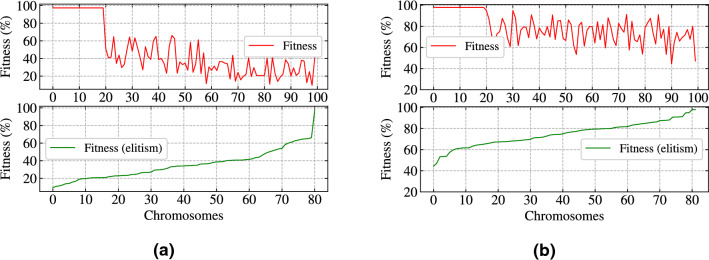
(**a**) The behaviour of fitness values without elitism (top) and unique fitness values after elitism (bottom) in case of Iris dataset. (**b**) The behaviour of fitness values without elitism (top) and unique fitness values after elitism (bottom) in case of WBC dataset.

Figure 1a (top), shows the mean accuracy (training set) for SpiFoG algorithm in case of Iris dataset having the value of *dt* as 1 ms where, it seems that the algorithm converges at 299th generation out of 1000 generations. Note that, up to 50 generations, accuracy (training set) increases rapidly and after that there is a slow change. This phenomenon demonstrates both exploitation and exploration of search space. Figure 1a (bottom), shows the search space exploration (i.e., big jumps) in case of Iris dataset and we refer this as unique or distinct change in accuracy (training set). When no distinct value occurs while training at a generation an algorithm is said to be stagnate at that value which justifies exploitation. All 30 generations shown in Fig. 1a (bottom) have a high variation in accuracy (training set) that depicts the fast convergence behaviour of the algorithm but still provides a better exploration with a population size 100. Figure 1b (top), shows the mean accuracy (training set) in case of WBC dataset having the value of *dt* as 1 ms where, it seems that the algorithm converges at 896th generation out of 1,000 generations. It is observed from Fig. 1b (top) that after 200 generation the change in accuracy (training set) is minute still it is allowed to evolve to settle down at a static value. Figure 1b (bottom) shows the search space exploration (i.e., big jumps) in case of WBC dataset where all 53 generations have a high variation in accuracy (training set). Figure 2c (top), shows the behaviour of fitness values without elitism for 80 chromosomes out of 100 (i.e., 20 best chromosomes are kept for the next generation and it is clearly visible in Fig. 2c (top)) in case of Iris dataset. The fitness values of non elitist chromosomes keeps increasing and decreasing at each generation. But, after elitism best chromosomes are combined with the chromosomes of the next generation results in a faster convergence, because best solutions never lost. Figure 2c (bottom) shows, unique or distinct values of fitness (exploration) after elitism in case of Iris dataset where the value of fitness only raises upwards which always guarantees the selection of parents having best fitness values for the next generation. Similarly, Fig. 2d (top) and (bottom) shows the behaviour of fitness values without elitist selection for 80 chromosomes in case of WBC dataset and distinct values of fitness (exploration) after elitism in case of WBC dataset.

**Figure 3:**
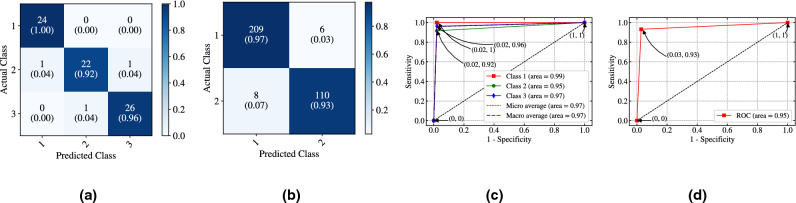
(**a**) Confusion matrix showing correctly classified and misclassified instances by the SpiFoG algorithm for Iris classification problem. (**b**) Confusion matrix showing correctly classified and misclassified instances by the SpiFoG algorithm for WBC classification problem. (**c**) ROC curve for the comparison of area under curve of each of the three classes of Iris dataset. (**d**) Single area under ROC curve for WBC dataset.

Figure 3a,b shows the confusion matrix for the Iris dataset and the WBC dataset where classified and misclassified samples are clearly visible for each dataset respectively. In addition, Fig. 3c,d shows the trade-off between true positive rate (sensitivity) and false positive rate (1-specificity) for every possible cut-off or threshold values in case of Iris and WBC dataset respectively. Note that, the ROC curve for Iris dataset shown in Fig. 3c, represents area of the individual classes where Class 1 shows the highest value in terms of area and both micro and macro average area is also shown as Iris is a multiclass classification problem. But, in case of WBC dataset (Fig. 3d), a single area under ROC curve is shown since it is a binary classification problem where the area is 0.95. The co-ordinates shown in Fig. 3c,d represents the vales of sensitivity vs. (1-specificity) for different threshold values. We have carried out the experiment using two values of *dt* one is 0.1 ms and other is 1 ms to compare with state-of-the-art algorithms specially in terms of CE. Table 1 shows the accuracy (training set and test set) for both the datasets where SpiFoG outperforms other algorithms in case of Iris dataset both in terms of accuracy (test set) and CE, the values are 97.24 ± 2.12 and 0.12$\times 10^{-3}$ respectively. Although DEPT-ESNN shows better accuracy (training set), it has a lower accuracy (test set). In case of WBC dataset, SpiFoG outperforms all other algorithms listed in Table 1 both in terms of accuracy (training set and test set) and CE. SpiFoG shows 98.32 ± 0.32 accuracy (training set), 97.92 ± 0.13 accuracy (test set) having value of *dt* as 0.1 ms but CE is found better with the value of *dt* as 1 ms in case of WBC dataset. In case of Iris dataset, SpiFoG shows a little bit lower accuracy (training set and test set) with *dt*=0.1 ms than *dt*=1 ms but, in case of WBC dataset, with *dt*=0.1 ms, it shows better accuracy (training set and test set). However, with *dt*=1 ms, both the dataset shows better CE. Note that, since one of our objective is to attain the better CE, we did not experiment with *dt*=0.01 ms as in other algorithms shown in Table 1, because lower values of *dt* takes more iterations (in this case a neuron fires lately) and thereby it shows lower value of CE.

## Methods

The primary objective of the proposed algorithm SpiFoG is to train a multilayer feed forward SNN efficiently. The encoding of real valued input features is carried out using the time-to-first-spike encoding scheme^8^. The LIF spiking neuron with an additional term random synaptic delay, and a double decay kernel function is given in Eqs. (4), (5), and (6). Both weights and delays are optimised using SpiFoG algorithm.

### Encoding of inputs and outputs

Using time-to-first-spike encoding scheme discussed in^8^, each real valued feature is converted to a number of overlapping Gaussian functions called encoding neurons having centred at $\mu$ as given in Eq. (2) and a spread $\sigma$ as given in Eq. (3). Thus a single input feature value is converted to *L* different response values at the range of [0, 1]. Then this responses are linearly converted to spike times within an encoding interval $\Delta T$ of [0, 10] ms where each spike times are the rounded spike times to its nearest time step *dt*. The value of *dt* was taken as 1 ms.

2 $$\begin{aligned} \mu _{i} = I_{min} + \left( \frac{2i-3}{2} \right) \times \left( \frac{I_{max}-I_{min}}{L-2} \right) \end{aligned}$$

where $I_{max}$ and $I_{min}$ are the maximum and minimum values of real valued feature, the values of i varies from 1 to *L*.

3 $$\begin{aligned} \sigma = \frac{1}{\beta } \times \left( \frac{I_{max}-I_{min}}{L-2} \right) \end{aligned}$$

where $\beta$ is the adjustment factor. The value of $\beta$ should be in the range [1, 2], but 1.5 is found to be experimentally best suited.

**Figure 4:**
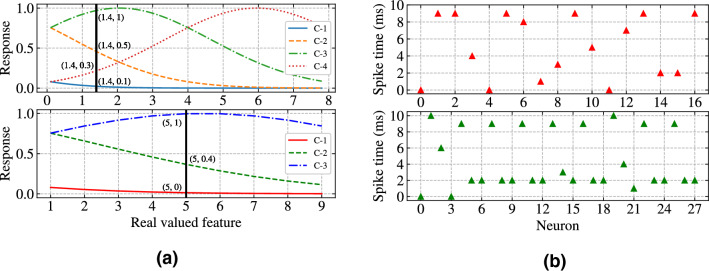
(**a**) The response values of one out of four real valued features of Iris dataset from the four overlapping Gaussian response functions (top) and the response values of one out of nine real valued features of WBC dataset from the three overlapping Gaussian response functions (bottom). (**b**) Raster plot of spike times for 16 input neurons and one bias neuron in case of Iris dataset (top) and raster plot of spike times for 27 input neurons and one bias neuron in case of WBC dataset (bottom).

Figure 4a (top) and (bottom) shows the conversion of real valued features of the Iris dataset and WBC dataset to their respective spike times using Gaussian overlapping response functions respectively. Figure 4b (top) and (bottom) shows the raster plot of 17 input neurons with their corresponding spike times in case of Iris dataset and the raster plot of 28 input neurons with their corresponding spike times in case of WBC dataset respectively. Spike times for bias neuron is set to 0 ms in case of both the datasets. Since the value of *L* is selected as 4 in case of Iris dataset, there are 4 Gaussian functions and the vertical line denotes one real valued feature as shown in Fig. 4a (top), cuts the Gaussian curves to yield the response values those are to be converted linearly within range [0, 10] ms. Similarly, in case of WBC dataset, selected $L=3$ encoding neurons are intersected by the real valued feature to yield response values as shown in Fig. 4a (bottom). The output spike times are restricted to 13 ms so that the total simulation time can be minimised. Three classes in case of Iris dataset are represented by 11 ms, 12 ms, and 13 ms and two classes in case of WBC dataset are represented by 11 ms, and 12 ms (trial and error basis).

### Proposed neuron model

The dynamics of spiking neurons is described by simple LIF neuron model^13, 14^. In this paper, the LIF neuron model having random synaptic delays is introduced which uses a double decaying synaptic model. The dynamics of the sub-threshold regime of a post-synaptic LIF neuron *j* is characterised in the Eq. (4).

4 $$\begin{aligned} \tau _{m}\frac{d\nu _{j}(t)}{dt} = -\nu _{j}(t-1) + \xi (t) R_{m} \end{aligned}$$

where $d\nu _{j}(t)$ is the small change in membrane potential of a post-synaptic neuron *j* at time *t*, *dt* is the unit time step, $R_{m}$ is the membrane resistance, $\tau _{m}$ is the membrane time constant, $\nu _{j}(t-1)$ is the membrane potential of post-synaptic neuron *j* at time $t-1$ (when $t=1$ ms, it becomes the resting potential of that post-synaptic neuron *j*), and $\xi (t)$ is the input stimuli at the post-synaptic neuron *j* from pre-synaptic neurons *i* given in Eq. (5). The value of $R_{m}$ is taken as 100 k$\Omega$.

5 $$\begin{aligned} \xi (t) = \sum _{j=1}^{N}\sum _{i=1}^{M}w_{ij} \Psi (t-t_{i}-\kappa _{ij}) \end{aligned}$$

where *M* is the total number of pre-synaptic neurons and *N* is the total number of post-synaptic neurons, $w_{ij}\in {\mathbf {R}}$ is the synaptic weights which describe the connection strength between two neurons *i* and *j*, $t_{i}$ denotes the spike time of pre-synaptic neuron *i*, and $\kappa _{ij} \in {\mathbf {I}}$ represents random synaptic delays between the synapses of neurons *i* and *j*. The definition of synaptic current kernel function for an input *x* is expressed as $\Psi (x)$ which is given in Eq. (6).

6 $$\begin{aligned} \Psi (x) = \left[ exp \left( -\frac{x}{\tau _{m}} \right) - exp \left( -\frac{x}{\tau _{s}} \right) \right] {\mathbf {H}}(x) \end{aligned}$$

where $\tau _{s}~(0<\tau _{s}<\tau _{m})$ is another time constant called synaptic time constant, ${\mathbf {H}}(x)$ is the Heviside function. In Eq. (7) the definition of ${\mathbf {H}}(x)$ is given. The time constants $\tau _{s}$ and $\tau _{m}$ controls the position of the function $\Psi (x)$ at the top. The decay and rise of steepness of the function $\Psi (x)$ is controlled by $\tau _{s}$ and $\tau _{m}$ respectively.

7 $$\begin{aligned} {\mathbf {H}}(x)= {\left\{ \begin{array}{ll} 1, &{}\quad {\text {if }} x>0 \\ 0, &{}\quad {\text {otherwise}} \end{array}\right. } \end{aligned}$$

Finally, the membrane potential $\nu _{j}$ at time *t* of the post-synaptic neuron *j* is updated using Eq. (8).

8 $$\begin{aligned} \nu _{j}(t) = \nu _{j}(t-1) + d \nu _{j}(t) \end{aligned}$$

When the value of $\nu _{j}$ reaches the threshold potential $\vartheta$, the post-synaptic neuron *j* fires a spike and the spike time $t_{j}$ for neuron *j* is recoded. After firing a spike membrane potential resets to a reset potential in case of multiple spike firing scheme. In this paper, the first time when $\nu _{j}$ reaches $\vartheta$ is considered and each neuron is allowed to fire only once. The spike times of neuron *j* is described by ${\mathbf {S}}_{j}$ given in Eq. (9).

9 $$\begin{aligned} {\mathbf {S}}_{j} = \left\{ t_{j}: 1 \le j \le M \right\} = \left\{ t : \nu _{j}(t) \ge \vartheta \right\} \end{aligned}$$

In Eq. (5), the term $\kappa _{ij}$ in the synapse model of the LIF neuron is introduced to make the network more biologically plausible and trained along with $w_{ij}$ to tune these parameters for the production of a better set of output spike times those are more closer to the target spike times.

### Network topology

**Figure 5:**
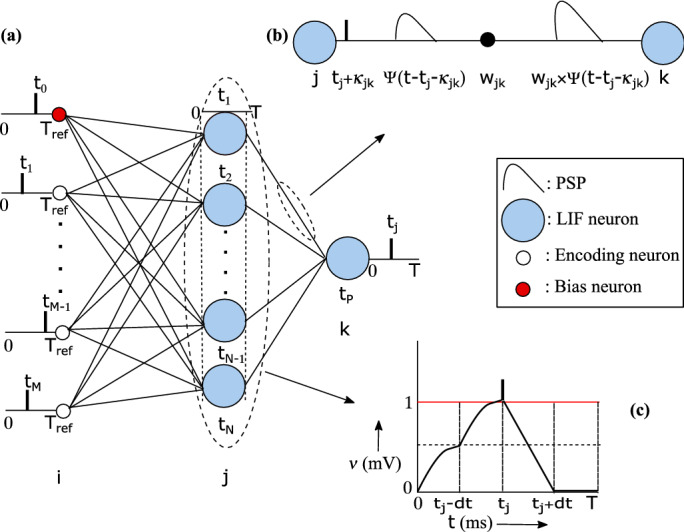
(**a**) The architecture of the multilayer feed forward SNN having *M* input spike times from $t_{1}$ to $t_{M}$, N hidden spike times from $t_{1}$ to $t_{N}$ produced when input spike times from $t_{1}$ to $t_{M}$ were supplied to LIF neuron along with $w_{ij}$ and $\kappa _{ij}$, and a single output spike time $t_{P}$. The input spike time $t_{0}$ is for the only bias neuron. (**b**) Synapse model where delay $\kappa _{jk}$ is associated with the weights $w_{jk}$ between pre-synaptic neuron *j* and post-synaptic neuron *k*. (**c**) The spiking activity of a post-synaptic neuron *j* after receiving input stimuli from pre-synaptic neurons *i* where $t_{j}$ is the spike time for post-synaptic neuron *j* when its membrane potential crosses the threshold value of 1 mV.

Figure 5a shows the network topology of the proposed multilayer feed forward SNN that consists of 17 input neurons, 12 hidden neurons, and 1 output neuron. The number of hidden neurons are selected on the basis of trail-and-error and only one hidden layer is used. Figure 5b,c show the synapse model where synaptic delay between pre-synaptic neuron *j* and post-synaptic neuron *k* denoted as $\kappa _{jk}$ is associated with the spike times $t_{j}$, and the change in membrane potential upon receiving the input stimuli which uses a double decay kernel function, respectively. The amplitude of $\Psi (x)$ increases with the increase in the value of synaptic weights. The more the value of synaptic weights more rapidly the amplitude increases. Therefore, the value of weights should be sufficient enough to reach the threshold value, otherwise the post-synaptic neurons end up with no spike times due to no firing. The negative weight values decreases the amplitude of kernel rather than increasing.

### Proposed learning method

The purpose of the proposed algorithm SpiFoG is to train the SNN architecture shown in Fig. 5a by optimising the initially generated random parameters such as synaptic weights *w* and synaptic delays $\kappa$. The optimised weights and delays forces the neurons to fire in a more sophisticated manner and then the firing spike times from hidden neurons acts as pre-synaptic spike times for the output neuron. The objective is to minimise the difference between actual spike times $t_{k}$ and desired spike times $t_{d}$. After the generation of actual spike times for all instances at *g*th generation $t^{g}_{k}$, where there are few spike times equal to the desired spike times $t_{d}$, the value of *w* and $\kappa$ are adjusted to produce more fine tuned spike times at $(g+1)$th generation $t^{g+1}_{k}$, using SpiFoG. The training continues until $t_{k}$ very closely converges to $t_{d}$. Firstly, upon receiving the inputs, the PSP of the hidden neurons changes. When the value of PSP of a hidden neuron *j* crosses the threshold $\vartheta$ at time *t* then the neuron *j* fires a spike at time *t*. The value of threshold $\vartheta$ is taken as 1 mV and the value of resting membrane potential $\nu _{r}$ is taken as 0 mV at time $t = 0$ ms. It is essential to optimise $t_{k}$ to match $t_{d}$ i.e., $t_{k} = f(w_{jk}, \kappa _{jk}) = g(w_{ij}, \kappa _{ij}) = h(t_{j})$. The values of *w* and $\kappa$ are tuned by forming the objective function *f* i.e., the fitness value of chromosomes and it is inversely proportional to the sum squared error (SSE) denoted by *e* and later it is converted to mean squared error (MSE) by taking mean in case of all training instances. In Eq. (10), the evaluation of fitness function is shown as the objective function which is the accuracy of the training set.

10 $$\begin{aligned} f= \frac{1}{1 + e} \end{aligned}$$

The definition of *e* is given in Eq. (11) where, $t_{k_{i}}$ is the output spike times for the only output neuron for all training samples and $t_{d_{i}}$ is the desired spike times for all training samples.

11 $$\begin{aligned} e = \frac{1}{2} \sum ^{n}_{i=1} ( t_{k_{i}} - t_{d_{i}} )^2 \end{aligned}$$

The main goal of SpiFoG is to maximise *f* because maximising *f* will minimise *e*. It uses elitist floating point genetic algorithm. Elitism is done to retain the best chromosomes from the current generation in order to utilise in the next generation^43^. Elitist chromosomes do not participate in crossover operation. This process is done in the selection phase of GA where a portion of chromosomes is removed from the parent population selected for crossover. Thus it retains the best chromosomes and guarantees to produce better solution in the next generation $(g+1)$ even if the fitness of all chromosomes in the $(g+1)$th generation are weaker than its previous generation *g* since best solutions never lost in this manner. In addition, since floating point gene values are used in this research, the optimisation method used by SpiFoG is termed as elitist floating point genetic algorithm. We have used 20% elitist chromosomes from the total population size 100. Maximising *f* tunes *w* and $\kappa$ and thus the value of *e* decreases. Note that, decrease in *e* means SNN learns more precisely from the given training dataset. In this research, rank selection method is used to select fittest chromosomes. SpiFoG uses combination of two different crossover methods on total population to produce better chromosomes in the next generation in an efficient manner. Hybrid crossover includes single point crossover method^44^ and another crossover method proposed by Haupt et al.^45^. The advantage of the later crossover method is that it always produce feasible solutions and therefore it is used to produce better solutions in terms of synaptic weights *w*. The definition of this crossover method is given in Eq. (12). The total genes $(i+k)\times j + (i+k)\times j$ of each chromosome is divided into two parts; one part is for the genes of *w* and other part is for the genes of $\kappa$. Single point crossover is used upon the values of $\kappa$ so that the value of delays remain integer even after crossover and to prevent the drastic change in delays. Single point crossover just alters the gene positions by selecting a single crossover point. After the generation of new chromosomes, uniform mutation is carried out to add some diversity to the search space. The definition of uniform mutation is given in Eq. (12).

12 $$\begin{aligned} C1_{p_{pop}}&=   P1_{p_{pop}} - r_{1}*(P1_{p_{pop}}-P2_{p_{pop}}); \nonumber \\ C2_{p_{pop}}&=   P2_{p_{pop}} + r_{2}*(P1_{p_{pop}}-P2_{p_{pop}}); \nonumber \\ \mu _{c_{pop}}&=   lb + r_{3} * (ub-lb) \end{aligned}$$

where $r_{1}$, $r_{2}$, and $r_{3}$ are random numbers between [0, 1], $P1_{p_{pop}}, P2_{p_{pop}}$ are first and second set of selected parents for crossover respectively, $C1_{p_{pop}}, C2_{p_{pop}}$ are two new set of solutions after crossover from first and second parents, *lb* and *ub* are the lower bound and upper bound of the population, where mutation is to be carried out, and $\mu _{c_{pop}}$ represents the mutated solutions of the crossover solutions. The value of crossover rate and mutation rate was taken as 0.8 (i.e., 80% non-elitist selection of parents from total population) and 0.1, respectively. Training stops when SpiFoG reaches maximum generation. Algorithm 1 shows the mechanism of the adjustment of $w_{ij}$ and $\kappa _{ij}$ between input and hidden layer neurons. The same mechanism is followed for the neurons reside in the hidden layer and output layer where there is a change in dimension of weights and delays denoted by $w_{jk}$ and $\kappa _{jk}$. Algorithm 2 shows the evaluation of fitness values. 
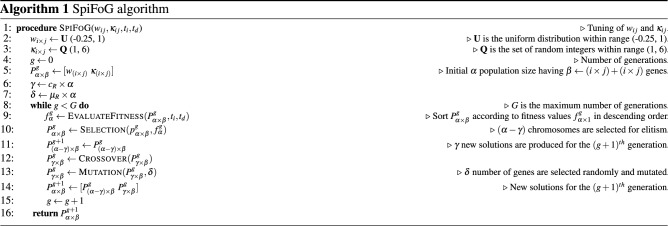
 
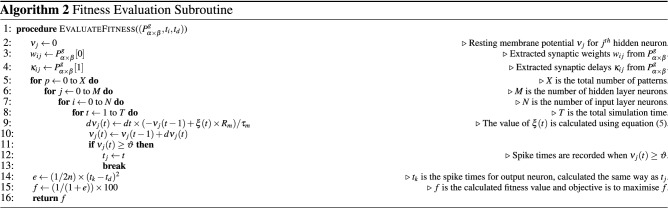


## Conclusion

This paper proposed SpiFoG algorithm to train multilayer feed forward SNN. It uses elitist floating point GA with hybrid crossover method discussed in this paper for fast convergence. The dynamics of the spiking neurons were described by LIF neuron introducing random synaptic delay. The delays are also tuned by the SpiFoG. The dimension of synaptic delay and synaptic weight were kept same to allow only a single synaptic terminal between any two pre-synaptic and post-synaptic neurons. To provide a better dimension in terms of biological plausibility both inhibitory and excitatory neurons were allowed. Moreover, the total simulation time was reduced to 20 ms which is directly responsible for better computational cost. SpiFoG shows best accuracy (test set) in case of Iris dataset which is 97.24% and best accuracy (98.32% training set and 97.92% test set) in case of WBC dataset. Also, best CE in case of both the datasets were observed for SpiFoG. The algorithms where CE could not be computed due to less information about the total simulation time, time step and convergence generation, those were compared with SpiFoG in terms of accuracy only. To summarise it is found that SpiFoG is computationally efficient as well as biologically plausible. However, SpiFoG allows the emission of only a single spike and therefore it can not be applied to SNNs where there it is more important to allow multiple spike times for each individual neuron.

It is possible to use other spiking neuron models to work with SpiFoG. For the future work we will use SpiFoG to classify time series data with different neuron model and evaluate the performance.
